# Combination of Abemaciclib following Eribulin Overcomes Palbociclib-Resistant Breast Cancer by Inhibiting the G2/M Cell Cycle Phase

**DOI:** 10.3390/cancers14010210

**Published:** 2022-01-01

**Authors:** Kamal Pandey, Nar Bahadur Katuwal, Nahee Park, Jin Hur, Young Bin Cho, Seung Ki Kim, Seung Ah Lee, Isaac Kim, Seung-Ryeol Lee, Yong Wha Moon

**Affiliations:** 1 Hematology and Oncology, Department of Internal Medicine, CHA Bundang Medical Center, CHA University, Seongnam 13488, Korea; pkamal@chauniv.ac.kr (K.P.); narbahadurkatwal@gmail.com (N.B.K.); skgml0413@naver.com (N.P.); hurjinz@naver.com (J.H.); ybyoungbin@naver.com (Y.B.C.); 2 Department of Biomedical Science, The Graduate School, CHA University, Seongnam 13620, Korea; 3 Department of Surgery, CHA Bundang Medical Center, CHA University, Seongnam 13620, Korea; mdsky@cha.ac.kr (S.K.K.); mdseungah@chamc.co.kr (S.A.L.); isaac24@cha.ac.kr (I.K.); 4 Department of Urology, CHA Bundang Medical Center, CHA University, Seongnam 13620, Korea

**Keywords:** CDK4/6, hormone receptor-positive breast cancer, drug resistance, PLK1

## Abstract

**Simple Summary:**

Cyclin-dependent kinase (CDK) 4/6 inhibitors, in combination with endocrine therapies, are now the standard of care for patients with metastatic hormone receptor-positive/human epidermal growth factor receptor 2-negative breast cancer. Despite the effectiveness of CDK4/6 inhibitors, acquired resistance occurs in almost all cases. We developed and used a palbociclib-resistant preclinical model and studied the overcoming strategies, using FDA-approved chemotherapy in combination with a CDK4/6 inhibitor. We demonstrated that sequential abemaciclib treatment following eribulin-enhanced anti-tumor activity in vitro and in vivo on the CDK4/6 inhibitor-resistant cells by more effectively inhibiting the G2/M cell cycle phase. The sequential combination of abemaciclib following eribulin could be an effective treatment strategy in overcoming resistance to CDK4/6 inhibitors in HR-positive breast cancer.

**Abstract:**

Breast cancer remains a leading cancer burden among women worldwide. Acquired resistance of cyclin-dependent kinase (CDK) 4/6 inhibitors occurs in almost all hormone receptor (HR)-positive subtype cases, comprising 70% of breast cancers, although CDK4/6 inhibitors combined with endocrine therapy are highly effective. CDK4/6 inhibitors are not expected to cooperate with cytotoxic chemotherapy based on the basic cytotoxic chemotherapy mode of action that inhibits rapidly proliferating cells. The palbociclib-resistant preclinical model developed in the current study investigated whether the combination of abemaciclib, CDK4/6 inhibitor with eribulin, an antimitotic chemotherapy could be a strategy to overcome palbociclib-resistant HR-positive breast cancer. The current study demonstrated that sequential abemaciclib treatment following eribulin synergistically suppressed CDK4/6 inhibitor-resistant cells by inhibiting the G2/M cell cycle phase more effectively. The current study showed the significant association of the pole-like kinase 1 (PLK1) level and palbociclib resistance. Moreover, the cumulative PLK1 inhibition in the G2/M phase by each eribulin or abemaciclib proved to be a mechanism of the synergistic effect. The synergistic antitumor effect was also supported by in vivo study. The sequential combination of abemaciclib following eribulin merits further clinical trials to overcome resistance to CDK4/6 inhibitors in HR-positive breast cancer.

## 1. Introduction

Breast cancer is the most common malignancy and a leading cause of cancer mortality among women globally [1]. There are three breast cancer subtypes based on the status of hormone receptor (HR) and human epidermal growth factor receptor 2 (HER2), i.e., HR-positive, HER2-positive, and triple-negative breast cancer (TNBC) [2]. HR-positive breast cancer represents the most frequent breast cancer subtype, and cyclin dependent kinase 4 and 6 (CDK4/6) inhibitor combined with aromatase inhibitors or tamoxifen is the current standard frontline therapy to treat such cancer types and CDK4/6 inhibitors combined with fulvestrant is a preferred second line therapy for the endocrine-resistant breast cancer patients [3]. After CDK4/6 inhibitor failure, another endocrine-based therapy, including fulvestrant or combined exemestane and everolimus, may be given to patients without visceral crises. Otherwise, cytotoxic chemotherapy may be an option for patients with visceral crises or endocrine refractoriness [4]. However, there is no established standard treatment in the CDK4/6 inhibitor resistance setting [5], suggesting the need to investigate resistance mechanisms and therapeutic strategies to overcome CDK4/6 inhibitor resistance.

Various mechanisms of resistance to CDK4/6 inhibitors have been previously explored [5]. In addition to RB loss and cyclin E overexpression, which were clinically confirmed mechanisms of resistance to CDK4/6 inhibitors in the literature [6,7,8], other various bypass signaling pathways associated with resistance mechanisms (e.g., activation of CDK2-cyclin E signaling [9] and growth signaling pathways) are also studied [10,11]. Those bypass signaling pathways, helping cells progress to the G2/M phase, losing their dependency on cyclin D-CDK4/6 signaling [5,9]. Thus, targeting the G2/M cell cycle phase could be a rational approach to inhibit the cells that pass the G1/S phase. Therefore, new effective combination therapy was tried by combining Food and Drug Administration (FDA)-approved anticancer drugs in palbociclib failure.

Eribulin is an FDA-approved chemotherapeutic drug, popularly used to treat metastatic breast cancer (MBC) patients who have previously received anthracycline- and/or taxane-based regimens [12]. Eribulin suppresses mitosis by directly binding to microtubule ends, inhibiting microtubule growth and tubulin aggregate formation [13]. This leads to the effective, irreversible mitotic block at the G2/M cell cycle phase, resulting in apoptosis [14]. Various clinical and preclinical studies have advocated the promising role of eribulin in breast cancer treatment [14,15]. Combining eribulin with CDK4/6 inhibitor could be an effective treatment strategy in overcoming resistance to CDK4/6 inhibitors, specifically to block the escaped cells that pass the G1/S cell cycle phase irrespective of the CDK4/6 inhibitor treatment. Based on these rationales, the current study investigated the potential synergism of combined eribulin and CDK4/6 inhibitor in a palbociclib-resistant breast cancer setting.

## 2. Materials and Methods

### 2.1. Drugs

Palbociclib (CDK4/6 inhibitor; Pfizer) was provided by Pfizer Inc. (Peapack, NJ, USA). Moreover, eribulin was provided by Eisai Co., Ltd. (Bunkyo City, Tokyo, Japan). Abemaciclib (CDK4/6 inhibitor; Eli Lilly and Company) and volasertib (PLK1 inhibitor) were purchased from ChemScene LLC. (Middlesex County, NJ, USA). All the drugs were dissolved in distilled water or dimethyl sulfoxide (Sigma-Aldrich, St. Louis, MO, USA). The drug concentration used in this study was not higher than the plasma concentration of the drugs applied to the patients [16,17,18] (Supplementary Table S1). IC50 concentration of each drug was calculated, and the cells were treated with either IC25 or IC50 concentration for all experiments.

### 2.2. Cancer Cell Lines

MCF7, T47D, and MDA-MB-231 cell lines were obtained from American Type Culture Collection (Manassas, VA, USA). Palbociclib-resistant MCF7 and T47D cells (MCF7-PR and T47D-PR) cells were generated in the laboratory of the current study, as previously mentioned [9]. MCF7, T47D, and MDA-MB-231 cells were routinely maintained in RPMI 1640 medium (WelGENE Inc., Daegu, Korea) supplemented with 10% heat-inactivated fetal bovine serum (cat# S001-01; WelGENE Inc.) and 1% 100× penicillin/streptomycin solution (WelGENE Inc.). MCF7-PR and T47D-PR cells were maintained with 1.5 µM palbociclib concentration, and the drug was washed out for 24–48 h before experiments were performed.

### 2.3. Cell Viability Assay

Cell viability was measured using the thiazolyl blue tetrazolium bromide (MTT; Sigma, St. Louis, MO, USA) assay as previously described [9]. In brief, 1000–2000 cells per well were seeded in a 96-well plate, allowed to attach overnight, and then treated with various concentrations of eribulin and abemaciclib or volasertib. For combination treatment, cells were treated with eribulin and abemaciclib either concomitantly or in a sequential treatment fashion, wherein cells were first treated with eribulin followed by abemaciclib at 24 h, 48 h, or without gaps. Cell viability was measured with the MTT assay. IC50 was determined using the CompuSyn software package (ComboSyn, Inc., Paramus, NJ, USA). In addition, the Chou–Talalay method [19] was used to calculate CI using the CompuSyn software package. CI < 1, CI > 1, and CI = 1 indicate synergism, antagonism, and additive effect, respectively.

### 2.4. Western Blot Analysis

Western blot analysis was done as previously described [9]. In brief, cells were lysed in the lysis buffer, and equal amounts of cell lysates were separated by sodium dodecyl sulfate–polyacrylamide gel electrophoresis and electrotransferred onto polyvinylidene difluoride membranes. The blot was then probed with primary antibodies followed by a reaction with horseradish peroxidase-conjugated secondary antibodies. Supplementary Table S2 presents a list of antibodies

### 2.5. Cell Cycle Assay

Cell cycle analysis was done as previously described [9]. The cells were harvested and washed twice with PBS. The cells were suspended in PBS with 50 and 100 µg/mL PI (Sigma-Aldrich) and RNase A (Sigma-Aldrich), respectively. The stained cells were then incubated in the dark at room temperature for 20 min. Cells were analyzed for DNA content using flow cytometry (Beckman Coulter CytoFLEX, Indianapolis, IN, USA), and the result of the cell cycle was analyzed by FlowJo software. The current study analyzed 10,000 cells/sample and determined the percentage of cells in each cell cycle phase.

### 2.6. Apoptosis Assay

An annexin V and PI (Sigma-Aldrich) stain was performed to determine the apoptosis levels after eribulin or abemaciclib treatment. All cells were harvested 48 h after drug treatment, washed in PBS, and resuspended with 400 µL annexin V 1 × binding buffer. Cells were stained with annexin V-APC and PI and incubated for 15 min at room temperature in the dark. Stained cells were washed twice with PBS and analyzed for apoptosis with flow cytometry. Moreover, 10,000 events were recorded using flow cytometry (Beckman Coulter CytoFLEX), and the proportion of apoptotic cells was analyzed.

### 2.7. CCLE Analysis

Cell line information, gene expression, and drug screening data were downloaded from the CCLE website, http://www.broadinstitute.org/ccle, accessed on 20 October 2021 (GSE36133) [20]. Using 38 breast cancer cell lines from the CCLE data, the expression of cell cycle-specific genes was correlated with palbociclib sensitivity, which was defined as an IC50 of ≤500 nM. Supplementary Table S3 presents a list of 38 breast cancer cell lines.

### 2.8. Public Gene Expression Profiling Datasets in Breast Cancer Patients

The current study used two independent public mRNA expression datasets of curatively resected HR-positive EBC (Supplementary Table S4) to validate that PLK1 mRNA expression level is associated with the prognosis in HR-positive breast cancer patients. Datasets (GSE26971 [21] and GSE2034 [22]) are mRNA microarray data. Series matrix files that the original authors already normalized were downloaded for analyses of the current study.

### 2.9. In Vivo Efficacy Studies in Xenograft Tumor Models

All animal procedures complied with the policies of the Institutional Animal Care and Use Committee (IACUC-190098) of CHA University, Gyeonggi-do, South Korea. Four-week-old female BALB/c nude mice were purchased from Orient Bio Inc. (Seongnam, Korea) and housed in a controlled environment at 25 °C on a 12-h light/dark cycle. Moreover, 1 × 10^7^ MCF7-PR cells suspended in Matrigel (Corning Matrigel, Corning, NY, USA) were subcutaneously inoculated into the mammary fat pad of the mouse. Estrogen valerate (3 μg/mouse) was subcutaneously injected every week as an estrogen supplement. Mice with established tumors of approximately 100 mm^3^ volumes were randomized into control and treatment groups. Eribulin was intraperitoneally injected once a week (1 mg/kg), whereas abemaciclib was administered by oral gavage 4 days a week (75 mg/kg) for 4 weeks. Here, abemaciclib was not given the day before or the day of eribulin administration, and eribulin was not administered <48 h after the last abemaciclib dosing, allowing sufficient time for the G1/S cell cycle blockage by abemaciclib to recover. Tumor volumes (measured with calipers and calculated using the formula: tumor length × tumor width 2 × 0.5) and animal body weight were recorded thrice a week for the study duration. After 28 days, all mice were sacrificed following experimental animal guidelines, and the xenografted tumors were excised and preserved for further analysis.

### 2.10. Statistical Analysis

Student’s *t*-test was performed to compare the two groups in the qRT-PCR and cell apoptosis assay. The correlations between gene expression and palbociclib sensitivity in the CCLE data were analyzed using the Pearson correlation coefficient. Distant recurrence-free survival (DRFS) was defined as the time from curative surgery to recurrence in distant organs or the last date that the patient was known to be free of distant recurrence (censoring time). DRFS was measured using the Kaplan–Meier estimator. In the public gene expression profiling datasets, the optimal cutoff was selected as the quartile with the minimum log-rank *p*-value in RFS analysis to divide patients into two groups of high or low PLK1 mRNA expression. The statistical significance of comparisons of survival curves was calculated by log-rank test. All statistical analyses were performed using SPSS version 19.0 (IBM SPSS Statistics 19.0, Armonk, NY, USA) except for the Pearson correlation coefficient, performed using GraphPad Prism (version 5.01; GraphPad Software, Inc. San Diego, CA, USA). All *p*-values were two-sided, and *p*-values < 0.05 were considered statistically significant.

## 3. Results

### 3.1. Eribulin Combined with CDK4/6 Inhibitor Enhances Cell Apoptosis in Palbociclib-Resistant Breast Cancer Cells

**Figure 1:**
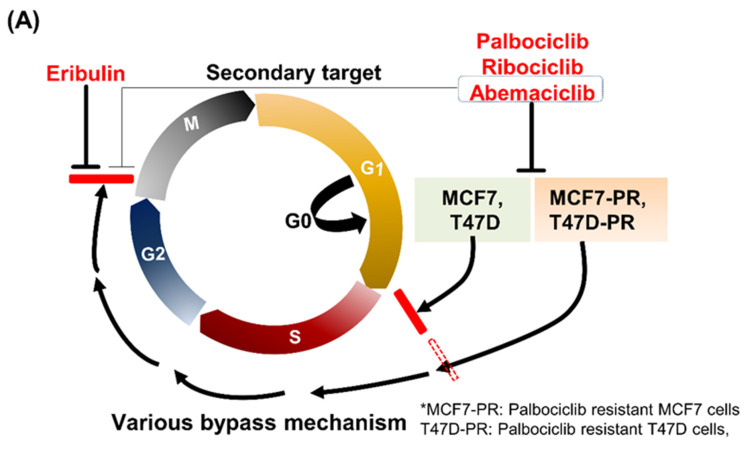

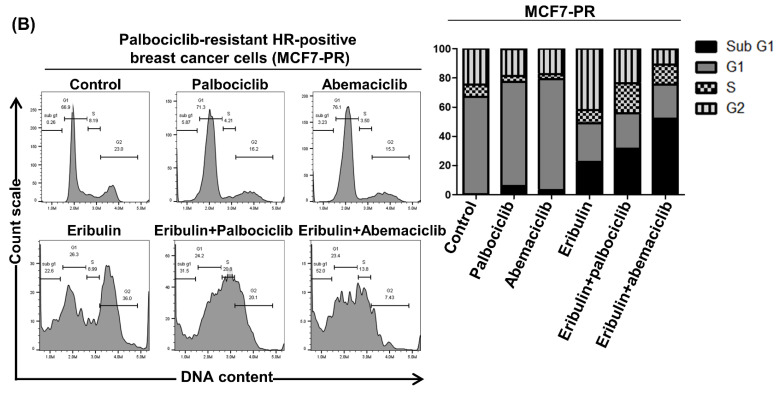
(**A**) Schematic figure demonstrating the escaping palbociclib-resistant cells, MCF7-PR and T47D-PR, via various bypass mechanisms. Mitosis of the aforementioned cells could be blocked by eribulin at the G2/M phase. (**B**) MCF7-PR cells were treated with IC25 concentration of eribulin, palbociclib, or abemaciclib and their combinations for 24 h. Eribulin and palbociclib or eribulin and abemaciclib combinations treatment was sequentially performed without a time gap between the drugs. Cell cycle distribution comparing the efficacy of palbociclib to abemaciclib after combination with eribulin was analyzed by flow cytometry. HR hormone receptor.

As previously described, palbociclib-resistant cell lines, MCF7-PR and T47D-PR, were successfully established from the HR-positive breast cancer cells MCF7 and T47D (Supplementary Table S5) [9]. CDK4/6 inhibitors were previously reported not to arrest palbociclib-resistant cells at the G1/S phase compared with palbociclib-sensitive cells [9]. Those cells not arrested at the G1/S phase could progress to the G2/M phase via various bypass mechanisms. Therefore, the current study investigated if eribulin, a mitotic inhibitor, could block those escaped cells at the G2/M phase (Figure 1A). Regarding CDK4/6 inhibitor selection to combine with eribulin, the current study compared the various profiles of three FDA-approved CDK4/6 inhibitors (i.e., palbociclib, ribociclib, and abemaciclib). Abemacilib has the lowest neutropenia incidence (21%), which may be beneficial in combining bone marrow-suppressing chemotherapy, although grade 3 diarrhea is more common with abemaciclib compared with palbociclib [23]. Abemaciclib also has a shorter half-life (18.3 h) than palbociclib and ribociclib (Supplementary Table S6) [23,24,25,26], which suggests that this cell cycle inhibitor may least hinder the cytotoxic chemotherapy action. Moreover, in cell cycle analysis of MCF7-PR cells, abemaciclib showed higher cell arrest at the G1 phase than palbociclib. Interestingly, abemaciclib was more effective in killing cells than palbociclib when combined with eribulin (Figure 1B). Lastly, despite being a retrospective design, a recent clinical study supported that abemaciclib would be effective after disease progression on prior CDK4/6 inhibitors treatment in HR-positive MBC patients [27]. Based on these results, the current study decided to use abamaciclib in combination with eribulin for all in vitro and in vivo experiments.

### 3.2. Sequential Eribulin and Abemaciclib Treatment Inhibits Breast Cancer Cell Viability

**Figure 2:**
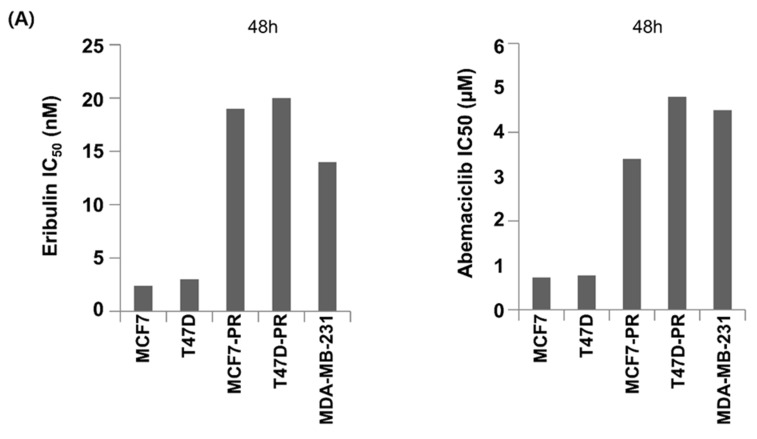

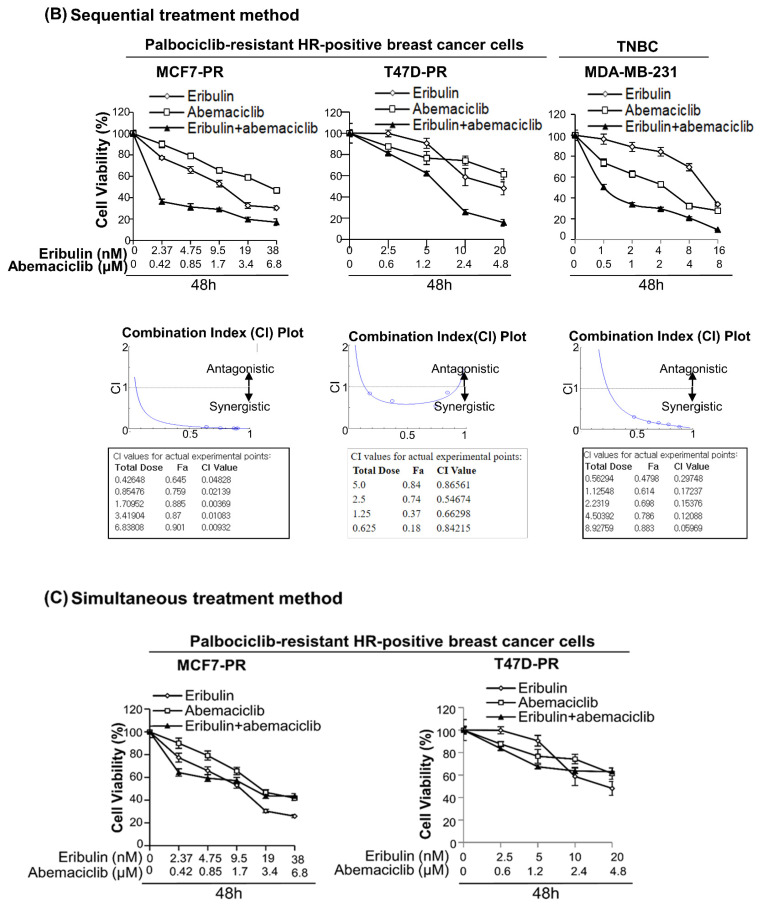

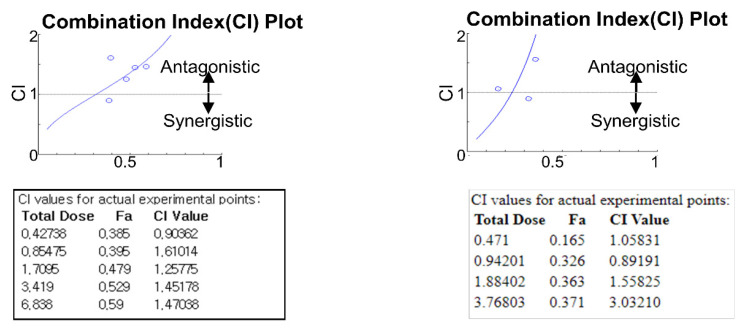
Sequential eribulin and abemaciclib treatment results in enhanced cell growth inhibition. (**A**) The viability of palbociclib-sensitive HR-positive cells (MCF7 and T47D), palbociclib-resistant HR-positive cells (MCF7-PR and T47D-PR), and TNBC (MDA-MB-231) cells after eribulin or abemaciclib treatment at different concentrations for 48 h were assessed by MTT. IC50 values were calculated using CompuSyn. The data represent the mean ± standard deviation of four replicates. (**B**) Cells were treated with increasing concentrations of eribulin and abemaciclib combinations at a fixed ratio for 48 h. Eribulin was given first, and abemaciclib was then given sequentially without a time gap, and cell viability was determined by MTT assay. (**C**) Cells were treated with increasing concentrations of eribulin and abemaciclib combinations at a fixed ratio for 48 h. Both drugs were given simultaneously, and the cell viability was determined by MTT assay. The CI was calculated by the Chou–Talalay method. CI < 1, CI > 1, and CI = 1 indicate synergism, antagonism, and additive effect, respectively. HR Hormone receptor, TNBC triple-negative breast cancer.

The current study first evaluated the antiproliferative activity of eribulin or abemaciclib in several breast cancer cell lines, including HR-positive palbociclib-sensitive, HR-positive palbociclib-resistant, and TNBC cells. Cells were treated with serial eribulin or abemaciclib dilutions, and cell viability was determined 48 h later. The half-maximal inhibitory concentration (IC50) of each drug was then calculated (Figure 2A).

Whether the combination of eribulin and abemaciclib resulted in more effective antiproliferative activity was next evaluated. Thus, multiple drug effect analyses were performed to determine the nature of interactions between eribulin and abemaciclib. Cells were treated with both agents either concomitantly or in a sequential treatment fashion, wherein cells were first treated with eribulin followed by abemaciclib at 24 h, 48 h, or without gaps. The drug combination strategy and its effect are shown in Figure S1. The sequential eribulin and abamaciclib treatment caused very strong synergism in all cell lines, including palbociclib-resistant and TNBC cells as well as palbociclib-sensitive cells (combination index [CI] < 1; Figure 2B and Figure S2). However, simultaneous treatment of both agents resulted in antagonistic interactions (CI > 1; Figure 2C). Among various sequential treatments, treatment with eribulin first followed by abemaciclib without treatment gap was applied thereafter in all experiments.

### 3.3. Sequential Eribulin and Abemaciclib Treatment Causes Mitotic Arrest Followed by Apoptosis

**Figure 3:**
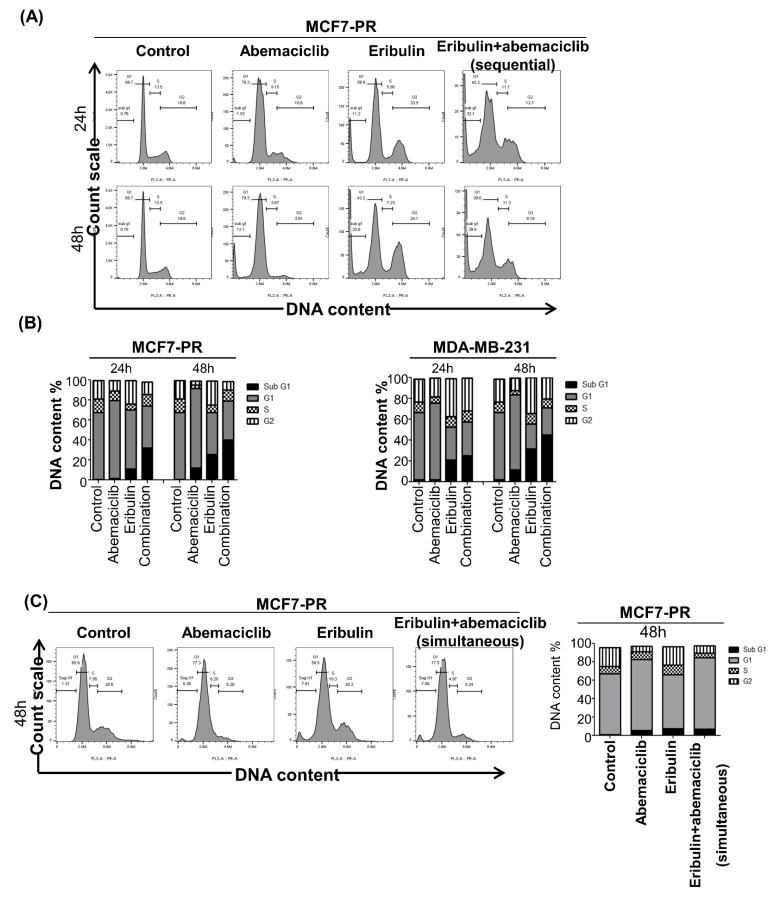

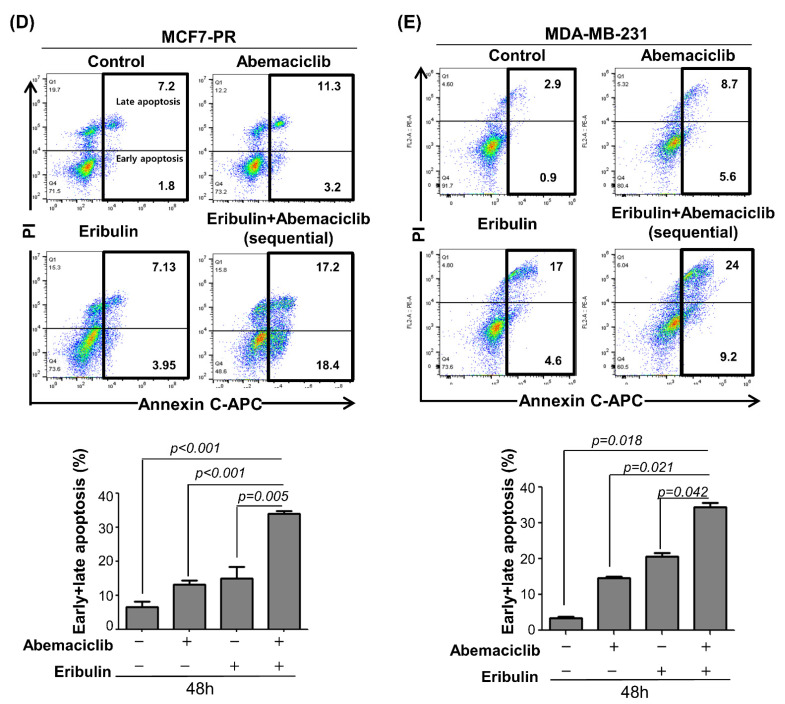
Eribulin and abemaciclib treatment causes mitotic arrest followed by cell death. (**A**) MCF7-PR and MDA-MB-231 cells were treated with IC25 concentration of eribulin or abemaciclib and their combination for 48 h. Eribulin and abemaciclib combinations treatment was sequentially performed without a time gap between the drugs. Cell cycle distribution was analyzed by flow cytometry. (**B**) The histogram represents the distribution of cells in the G0/G1, S, and G2/M phases, and the bar graph indicates the percentage of cells in the G0/G1, S, and G2/M phases of the cell cycle. (**C**) MCF7-PR cells were treated with IC25 concentration of eribulin or abemaciclib and their combination for 48 h. Both drugs were given simultaneously for the combination treatment group. (**D**,**E**) MCF7-PR and MDA-MB-231 cells were treated with IC25 concentration of eribulin or abemaciclib and their combination for 48 h. Eribulin and abemaciclib combination treatment were performed sequentially, without a time gap between the drugs. Cells were stained with annexin V-APC/PI, and the apoptosis was measured by flow cytometry. The data shown are representative of three independent experiments. *p*-values were calculated by Student’s *t*-test. Histograms are drawn from the summation of the numbers in the box drawn. Data are presented as mean ± standard deviation from three independent experiments.

The current study next examined the effect of combination treatment on the cell cycle to elucidate the synergistic mechanism of eribulin plus abemaciclib in breast cancer cells. Eribulin is known to induce G2/M arrest [28], while abemaciclib induces G1 arrest in cancer cells [29]. Palbociclib-resistant cells, MCF7-PR, were treated with eribulin with or without abemaciclib quarter-maximal inhibitory concentration (IC25) concentrations for 48 h, and cell cycle was analyzed by flow cytometry. As expected, cells were arrested at the G2/M and G1 phases after treatment with eribulin and abemaciclib as a single agent, respectively. Notably, eribulin combined with abemaciclib treatment dramatically induced the sub-G1 phase, indicating synergistic cytotoxic activity (Figure 3A). In addition, we observed higher higher G2/M arrest at 12 h time point compared to 24 h and 48 h denoting prolonged G2/M arrest occurs early time point and this eventually triggers cell death by apoptosis (Figure S3A). Intriguingly, palbociclib-sensitive HR-positive cells (MCF7) and TNBC cells (MDA-MB-231) also showed a similar pattern (Figure 3B and Figure S3A,B). However, unlikely the sequential treatment of both drugs, simultaneous treatment fashion could not induce the sub-G1 in the cell cycle analysis (Figure 3C). This result indicated antagonistic activity between eribulin and abemaciclib by simultaneous treatment with both drugs. To further investigate whether the synergism of sequential eribulin and abemaciclib treatment was due to apoptosis, the current study performed annexin V/propidium iodide (PI) staining and found a significant increase in both early and late apoptosis rate in both palbociclib-resistant and sensitive cells and TNBC cells (Figure 3D,E and Figure S3C).

### 3.4. Combined Eribulin and Abemaciclib Treatment Induce Apoptosis by Inhibiting Pole-Like Kinase 1

**Figure 4:**
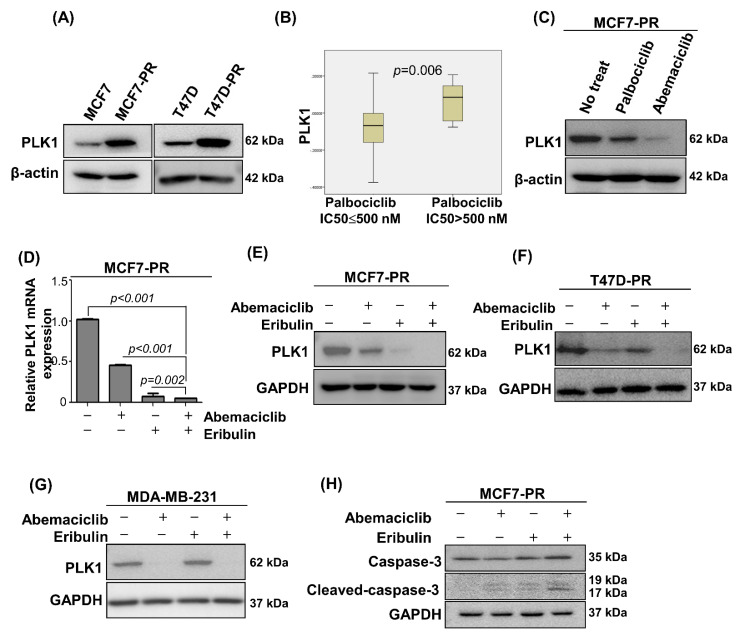

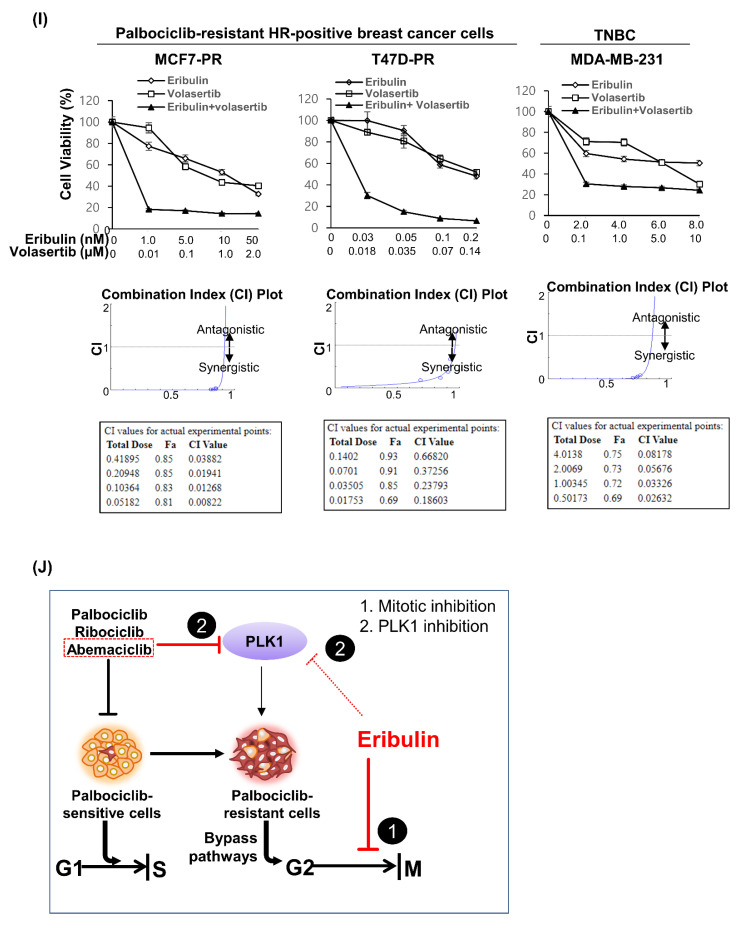
Eribulin synergizes abemaciclib via PLK1 inhibition in the G2/M phase. (**A**) PLK1 expression in palbociclib-resistant cells compared with their sensitive counterparts was analyzed by western blot. (**B**) Correlation of CCLE, PLK1 gene and palbociclib sensitivity, which was defined as IC50 ≤ 500 nM in breast cancer cell lines. *p*-value was calculated by Student’s *t*-tests. (**C**) MCF7-PR cells were treated with IC50 concentration of palbociclib or abemaciclib for 48 h. PLK1 expression was analyzed by western blot. (**D**) Relative mRNA expression determined by qRT-PCR. PLK1 in MCF7-PR cells was downregulated with IC50 concentration of eribulin or abemaciclib and more downregulated with their sequential combination. *p*-values were calculated by Student’s *t*-test. Data are presented as mean ± standard deviation from three independent experiments. (**E**–**H**) Cells were treated with IC50 concentration of eribulin or abemaciclib and their combination for 48 h. (**E**–**G**) PLK1 expression and (**H**) cleaved caspase-3 expression was analyzed by western blot. (**I**) The viability of palbociclib-resistant HR-positive cells (MCF7-PR and T47D-PR) and TNBC (MDA-MB-231) cells after treatment with eribulin or volasertib at different concentrations for 48 h was assessed by MTT. IC50 values were calculated using CompuSyn. Data represent the mean ± standard deviation of four replicates. (**J**) Schematic diagram showing the mechanism of eribulin and abemaciclib inhibiting the escaped cells at the G2/M phase. Full length blots (**A**,**C**,**E**–**H**) are presented in Figure S5.

The pole-like kinase 1 (PLK1) is a protein kinase that regulates the cell cycle during mitotic entry and the G2/M checkpoint [30,31]. Considering the multiple PLK1 functions in the G2/M phase, the current study next examined the association of PLK1 expression and palbociclib resistance, which was not previously well-studied [32]. The current study remarkably noticed the PLK1 overexpression on MCF7-PR and T47D-PR cells compared with their parental counterparts (Figure 4A). The TNBC cell line, MDA-MB-231 cells, also showed PLK1 overexpression compared with palbociclib-sensitive cells, MCF7 and T47D (Figure S4A). Furthermore, when analyzing the association of palbociclib activity and PLK1 mRNA expression in 38 breast cancer cell lines using Cancer Cell Line Encyclopedia (CCLE) data, the current study found that cells with low palbociclib activity (IC50 > 500 nM) had higher PLK1 expression (*p* = 0.006; Figure 4B). MCF7-PR cells treated with IC50 concentrations of palbocilib or abemaciclib had a higher PLK1 inhibition with abemaciclib than palbociclib treatment (Figure 4C), indicating the enhanced activity of abemaciclib in the G2/M phase. The current study then performed quantitative real-time polymerase chain reaction (qRT-PCR) and western blot analyses to detect the PLK1 changes after the sequential eribulin and abemaciclib treatment in palbociclib-resistant cells. The current study demonstrated that the mRNA and PLK1 protein levels decreased in the combination treatment cells compared with the eribulin or abemaciclib treatment alone or with no treatment control cells (Figure 4D–G). In addition, the downstream kinases of PLK1, were also inhibited by the combined treatment of eribulin and abemaciclib. (Figure S4D). Cleaved caspase-3 increased in the combination treatment cells compared with the monotherapy or no treatment cells, which implies that combination treatment enhances apoptosis (Figure 4H). This synergistic mechanism occurred in the palbociclib-resistant and TNBC cells as well as palbociclib-sensitive cells (Figure S4B,C).

The current study further evaluated whether the synergistic effect of combined eribulin and abemaciclib occurs via PLK1 inhibition in the G2/M phase by using a selective PLK1 inhibitor, volasertib [33]. The combined volasertib and eribulin treatment showed strong synergism in palbociclib-resistant and TNBC cells (CI < 1; Figure 4I). In addition, the current study performed cell cycle assay and annexin V/propidium iodide (PI) staining assay and found a significant increase in both early and late apoptosis rate in palbociclib-resistant and TNBC cells (Figure S4E,F). To sum up, abemaciclib inhibits the PLK1 as a secondary target at the G2/M phase and prolongs the eribulin-induced G2/M arrest more, leading to cell apoptosis (Figure 4J) in palbociclib-resistant cells.

The current study investigated the impact of PLK1 overexpression on disease prognosis in HR-positive early breast cancer (EBC) patients to demonstrate the PLK1 clinical relevance in HR-positive breast cancer. Two independent public mRNA expression datasets from HR-positive EBC (210 patients from GSE26971 and 209 patients from GSE2034) showed a trend toward an association of PLK1 overexpression and a higher risk of distant recurrence (Figure S4G). This trend suggests that PLK1 could be a potential direct or indirect therapeutic target in HR-positive breast cancer even though the gene expression analyses of the current study were derived from breast cancer cohorts that were not exposed to CDK4/6 inhibitors.

### 3.5. Combined Eribulin and Abemaciclib Treatment Significantly Suppresses Tumor Growth in a Palbociclib-Resistant Breast Cancer Xenograft Model

**Figure 5:**
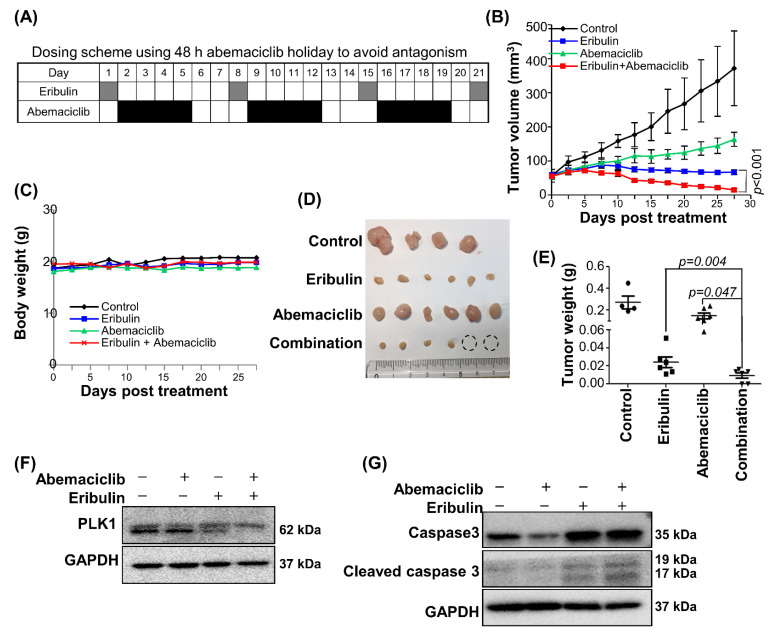
Combined eribulin and abemaciclib treatment enhances in vivo antitumor activity in palbociclib-resistant breast cancer xenograft. (**A**) Drug dosing scheme for the in vivo experimental procedure. (**B**) Mean tumor growth curve of MCF7-PR xenograft treated with eribulin, abemaciclib, or a combination of those two. Tumor volumes were monitored every 2–3 days. *p*-value was calculated with two-way analysis of variance after Bonferroni correction on day 28 after drug treatment initiation. Data are presented as the mean ± standard error of the mean (SEM). (**C**) The mice body weight graph indicated that drug treatment did not cause any bodyweight loss. (**D**) Xenografted tumors were harvested from each group of mice at the end of the experiment. Dotted circles indicated complete tumor regression. (**E**) The weights of the tumors were measured. *p*-values were calculated by Student’s *t*-test. Data are presented as the mean ± SEM. NS indicates not significant. All the student’s- *t*-test performed were two-tail and unpaired. (**F**,**G**) Western blot using MCF7-PR xenograft after 28 days of treatment showed a greater PLK1 suppression and cleaved caspase-3 induction in the combination group than eribulin or abemaciclib single-treatment groups. Full length blots (**F**,**G**) are presented in Figure S5.

The current study next investigated the in vivo efficacy of eribulin and abemaciclib or their combination in an MCF7-PR murine xenograft model. Mice were treated with 1 mg/kg eribulin once a week combined with or without 75 mg/kg abemaciclib once daily, 4 days a week for 4 weeks with 48-h abemaciclib holiday in the combination group (Figure 5A). The tumor growth rate was significantly suppressed by treatment with abemaciclib compared with vehicle, whereas antitumor activity was dramatically enhanced in the combination group (Figure 5B; *p* < 0.001), similar to an in vitro study. The combination treatment was well-tolerated, without causing significant body weight loss compared with the eribulin or abemaciclib alone group (Figure 5C). Strikingly, eribulin plus abemaciclib treatment led to complete tumor regression in two of six mice (33.3%; Figure 5D). The mice were sacrificed on day 28 after drug treatment initiation, the tumors were excised (Figure 5D), and their weights were analyzed (Figure 5E). The results obtained were similar to those based on the calculated tumor volumes. Western blot analysis of the tumor tissue lysates showed greater PLK1 inhibition and cleaved caspase-3 induction in the combination group than abemaciclib alone (Figure 5F,G).

## 4. Discussion

Most patients eventually develop disease progression despite effective anticancer CDK4/6 inhibitor activity in HR-positive/HER2-negative MBC treatment. The acquired resistance to these inhibitors has become an inevitable clinical issue, emphasizing the need to identify pharmacological targets that may block the consequent resistance onset. The present study developed and used a palbociclib-resistant preclinical model and studied the overcoming strategies, using FDA-approved chemotherapy in combination with a CDK4/6 inhibitor. The current study demonstrated that the combined eribulin and abemaciclib treatment might offer a new potential strategy to overcome CDK4/6 inhibitor resistance. Intriguingly, the current study could be the first to report that the combined cytotoxic chemotherapy and cell cycle inhibitor could be synergistic by adjusting the dosing schedule to avoid antagonism.

Researchers hardly consider combining cytotoxic chemotherapy and CDK4/6 inhibitors because CDK4/6 inhibitors should not cooperate with cytotoxic chemotherapies. After all, the former prevents cell cycle entry, thus interfering with the S phase or mitosis-targeting agents, based on cytotoxic chemotherapy’s basic mode of action, inhibiting rapid cell proliferation [34]. Furthermore, overlapping myelosuppression (e.g., neutropenia) is another concern when two myelosuppressing agents are combined. Some preclinical studies previously demonstrated the antagonism between CDK4/6 inhibitors and chemotherapeutic agents, where CDK4/6 inhibitors protected the cells from chemotherapeutic agents because both drugs were given simultaneously [35,36]. However, the sequential treatment of CDK4/6 inhibitor and chemotherapy synergistically potentiated the combination efficacy in other preclinical studies [34,37]. The current study evaluated the efficacy of combined abemaciclib and eribulin in palbociclib-resistant HR-positive and TNBC cells according to different treatment schedules (simultaneous versus sequential treatment). Thus, the current study demonstrated that sequential treatment strategy inhibited cell proliferation and induced cell death more efficaciously than single-agent treatment. Based on the literature and the current study’s findings, the drug treatment schedule is a critical aspect requiring careful consideration when planning CDK4/6 inhibition plus chemotherapy-based therapies. In a future clinical trial design, both drugs’ dosing schedules should be adjusted, and abemaciclib should be given sequentially after eribulin to induce synergism. Moreover, in terms of toxicity, patients should be monitered carefully for neutropenia, even though the least possibility of neutropenia is expected for abemaciclib as a combinatory.

Multiple resistance mechanisms to CDK4/6 inhibitors have been previously explained. In addition, we also previously demonstrated that immune pathway deregulation, as well as the RB loss and cyclin E-CDK2 pathway activation, were associated with palbociclib resistance [9,38]. Moreover, AURKA amplification [7], MDM2 amplification [39], PTEN loss [40], or upstream growth factor receptor signaling activation (e.g., FGFR [10] or PI3K/AKT/mTOR [11]) are also highlighted as the potential resistance mechanism to CDK4/6 inhibitors. Various bypass resistance mechanisms drive cells to escape CDK4/6 inhibition, and cells progress to S or G2/M cell cycle phase, which motivated us to study on the strategies to block cells that escape cyclin D-CDK4/6 signaling. The current study demonstrated that eribulin effectively killed the escaping cells passing through the cell cycle G1/S phase despite CDK4/6 inhibitor treatment. The PLK1 is a key regulator that drives cells to progress to the G2/M phase by controlling the CDK1/cyclin B complex activity and is also known to phosphorylate and regulate several cellular proteins during mitosis [41]. Various studies reported that the PLK1 overexpression is associated with cancer progression and drug resistance [30,42,43]. However, little is known about the association of PLK1 and CDK4/6 inhibitor resistance. This study first demonstrated the association of the PLK1 and palbociclib resistance by using the palbociclib-resistant preclinical model of the current study and CCLE data. Although palbociclib and abemaciclib are both CDK4/6 inhibitors, it is known that these agents can have different activity to inhibit various kinase enzymes [44]. Abemaciclib is involved in PLK1 inhibition at the G2/M phase as secondary target [45] in addition to the G1/S arrest. Moreover, abemaciclib was more potent in inhibiting PLK1 compared with palbociclib (Figure 4C). Based on these observations, abemaciclib was selected as a good eribulin combinator in the palbociclib-resistant preclinical model, where eribulin arrests cells at the G2/M phase and sequential abemaciclib treatment prolongs the G2/M arrest via PLK1 inhibition and sustaining mitotic blockade, consequently leading to apoptosis in HR-positive and TNBC cells.

Several ongoing phase I or II trials with taxanes (e.g., paclitaxel, docetaxel, or nab-paclitaxel) were noted in combination with CDK4/6 inhibitors (Supplementary Table S7). The combination of eribulin and abemaciclib has never been tested in clinical trials. Thus, the combination of eribulin and abemaciclib in the current study has advantages. First, the combination of the current study may benefit palbociclib-resistant HR-positive breast cancer patients based on a preclinical synergism shown in this study. None of the clinical trials of combined taxanes and CDK4/6 inhibitors listed in Supplementary Table S7 cover CDK4/6 inhibitor-resistant breast cancer settings. Second, eribulin has proved effective in taxane-pretreated patients with breast cancer from phase III trials (e.g., EMBRACE [46] and Study 301) [47]. Thereby, the combination of eribulin and abemaciclib of the current study may have anticancer activity in a resistance setting of both taxane and CDK4/6 inhibitor (e.g., palbociclib or ribociclib). Furthermore, the combination of eribulin and abemaciclib may be more widely useful than the taxane-based combination because taxane resistance will occur in most cases. Third, currently, several new agents, including CDK7 inhibitors, CDK2 inhibitors, selective estrogen receptor downregulator, BCL-2 inhibitor, FGFR inhibitor, immune checkpoint inhibitors, and others, are being tested in phase I–III clinical trials after CDK4/6 inhibitor progression [48]. However, the combination of eribulin and abemaciclib can be more promptly applied in practice if a clinical trial proves the efficacy of this combination because both drugs are already FDA-approved in HR-positive breast cancer patients. Collectively, the current study’s findings suggest the possibility of applying this combination for breast cancer treatment or as an overcoming strategy in palbociclib-resistant breast cancer cases.

## 5. Conclusions

In conclusion, the current study demonstrated that sequential abemaciclib treatment following eribulin enhanced anti-tumor activity in vitro and in vivo on the CDK4/6 inhibitor-resistant cells by more effectively inhibiting the G2/M cell cycle phase. This sequential combination of abemaciclib following eribulin merits further clinical trials to overcome resistance to CDK4/6 inhibitors in HR-positive breast cancer.

## Data Availability

Data available in a publicly accessible repository 3rd Party Data: Data sharing not applicable.

## Supplementary Materials

The following are available online at https://www.mdpi.com/article/10.3390/cancers14010210/s1, Figure S1: Drug treatment schemes in vitro and their outcome. Figure S2: Sequential eribulin and abemaciclib treatment results in synergistic cell growth inhibition in MCF7 and T47D cells treated with increasing concentration of eribulin and abemaciclib combinations at a fixed ratio. Figure S3: Eribulin and abemaciclib treatment causes mitotic arrest followed by cell death. Figure S4: Abemaciclib synergizes eribulin via inhibiting PLK1 in the G2/M phase. Figure S5: PDF file. Un-cropped immunoblot images. Table S1: Comparison of IC50 concentration of palbociclib-resistant cells with their parental counterparts. Table S2: Toxicity and half-life profile of CDK4/6 inhibitors. Table S3: Completed or ongoing clinical trials of combined taxanes and CDK4/6 inhibitor. Table S4: Primary antibodies used for western blot. Table S5: A list of 38 breast cancer cell lines in CCLE data base. Table S6: Patient characteristics in two public mRNA expression data sets
