# Supplementary material for: Combination of Abemaciclib following Eribulin Overcomes Palbociclib-Resistant Breast Cancer by Inhibiting the G2/M Cell Cycle Phase
Source: Cancers (Basel). 2022 Jan 1;14(1):210. doi: 10.3390/cancers14010210 (PMC8750394; doi:10.3390/cancers14010210)
Supplement: Supplementary file 1 [file cancers-14-00210-s001.zip › cancers-1456749-supplementary.pdf]

cancers

MDPI

*Article*

# Combination of Abemaciclib following Eribulin Overcomes Palbociclib-Resistant Breast Cancer by Inhibiting the G2/M Cell Cycle Phase

Kamal Pandey[1,2], Nar Bahadur Katuwal[1,2], Nahee Park[1], Jin Hur[1,2], Young Bin Cho[1], Seung Ki Kim[3], Seung Ah Lee[3], Isaac Kim[3], Seung-Ryeol Lee[4]* and Yong Wha Moon[1]*

## Supplementary figures

**S1**

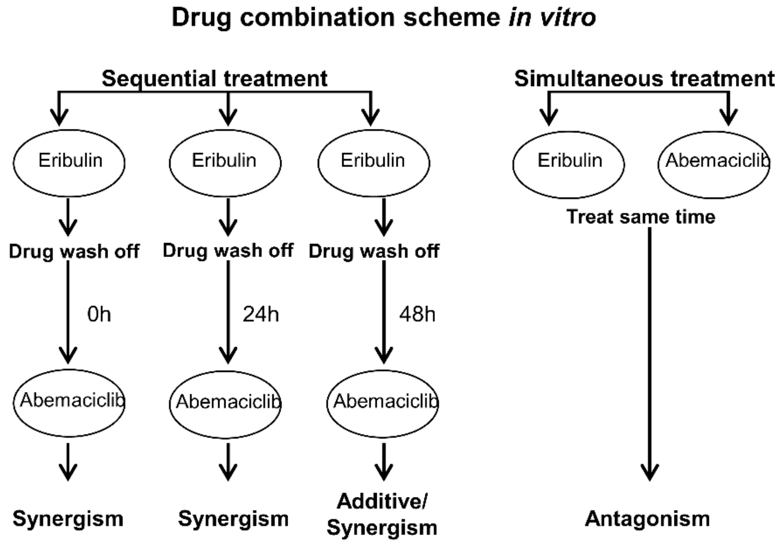


**Figure S1.** Drug treatment schemes in vitro and their outcome. The synergistic and/or antagonistic effect of drug combination depends upon eribulin and abemaciclib treatment sequence.

## S2 Sequential treatment method

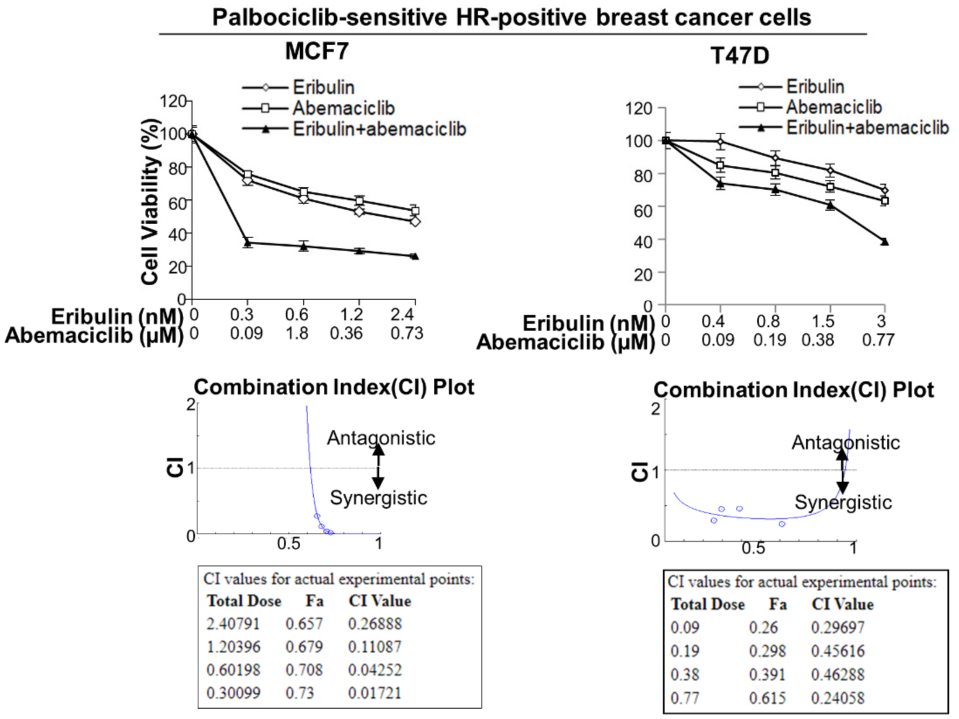


**Figure S2.** Sequential eribulin and abemaciclib treatment results in synergistic cell growth inhibition in MCF7 and T47D cells treated with increasing concentration of eribulin and abemaciclib combinations at a fixed ratio. Treatment was performed sequentially without a time gap between both the drugs. Cell viability was determined by MTT assay. CI was calculated by the Chou–Talalay method[41]. CI <1, CI >1, and CI = 1 indicate synergism, antagonism, and additive effect, respectively. (HR hormone receptor).

**S3**

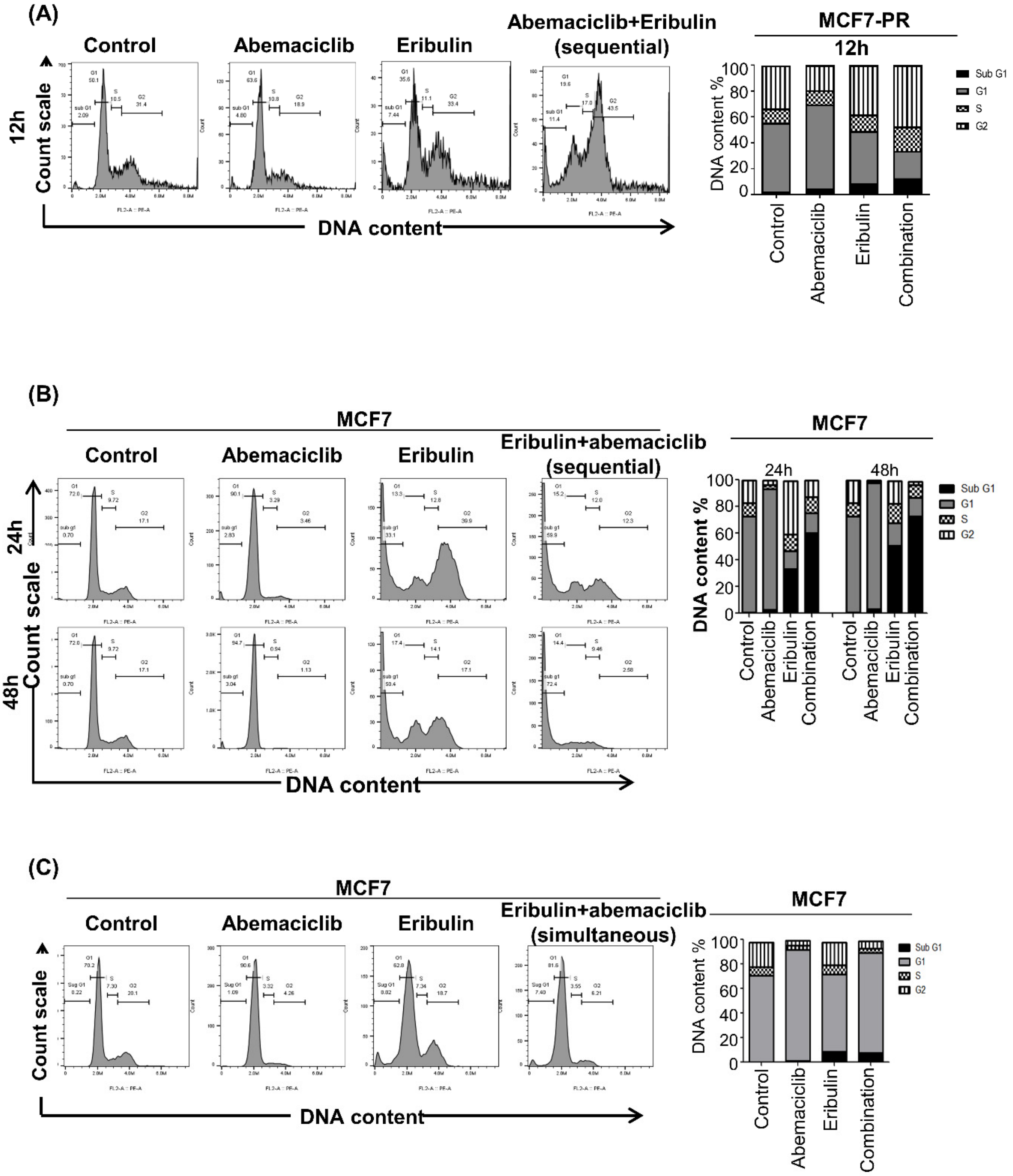
(A)
Control
Abemaciclib
Eribulin
Abemaciclib+Eribulin (sequential)
12h
Count scale
DNA content
MCF7-PR
12h
DNA content %
Sub G1
G1
S
G2
Control
Abemaciclib
Eribulin
Combination
(B)
MCF7
Control
Abemaciclib
Eribulin
Eribulin+abemaciclib (sequential)
24h
48h
Count scale
DNA content
MCF7
24h
48h
DNA content %
Sub G1
G1
S
G2
Control
Abemaciclib
Eribulin
Combination
(C)
MCF7
Control
Abemaciclib
Eribulin
Eribulin+abemaciclib (simultaneous)
Count scale
DNA content
MCF7
DNA content %
Sub G1
G1
S
G2
Control
Abemaciclib
Eribulin
Combination

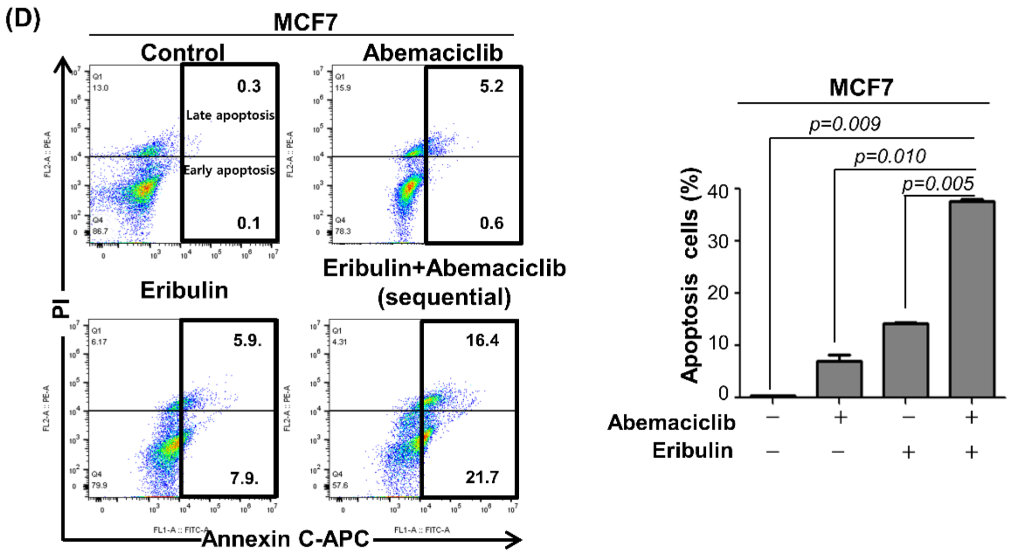


**Figure S3.** Eribulin and abemaciclib treatment causes mitotic arrest followed by cell death. (A) MCF7-PR cells were treated for 12h with IC25 concentration of eribulin or abemaciclib and their combination. Cell cycle distribution was analyzed by flow cytometry. (B-C) MCF7 cells were treated with IC25 concentration of eribulin or abemaciclib and their combination (B) sequentially without a time gap or (C) simultaneously for 48 h. Cell cycle distribution was analyzed by flow cytometry. (D) MCF7 cells were treated with IC25 concentration of eribulin or abemaciclib and their sequential combination for 48 h. Cells were stained with annexin V-APC/PI, and the apoptosis was measured by flow cytometry. The data shown are representative of three independent experiments. P-values were calculated by Student's t-test. Histograms are drawn from the summation of numbers in the box drawn. Data are presented as mean ± standard deviation from three independent experiments.

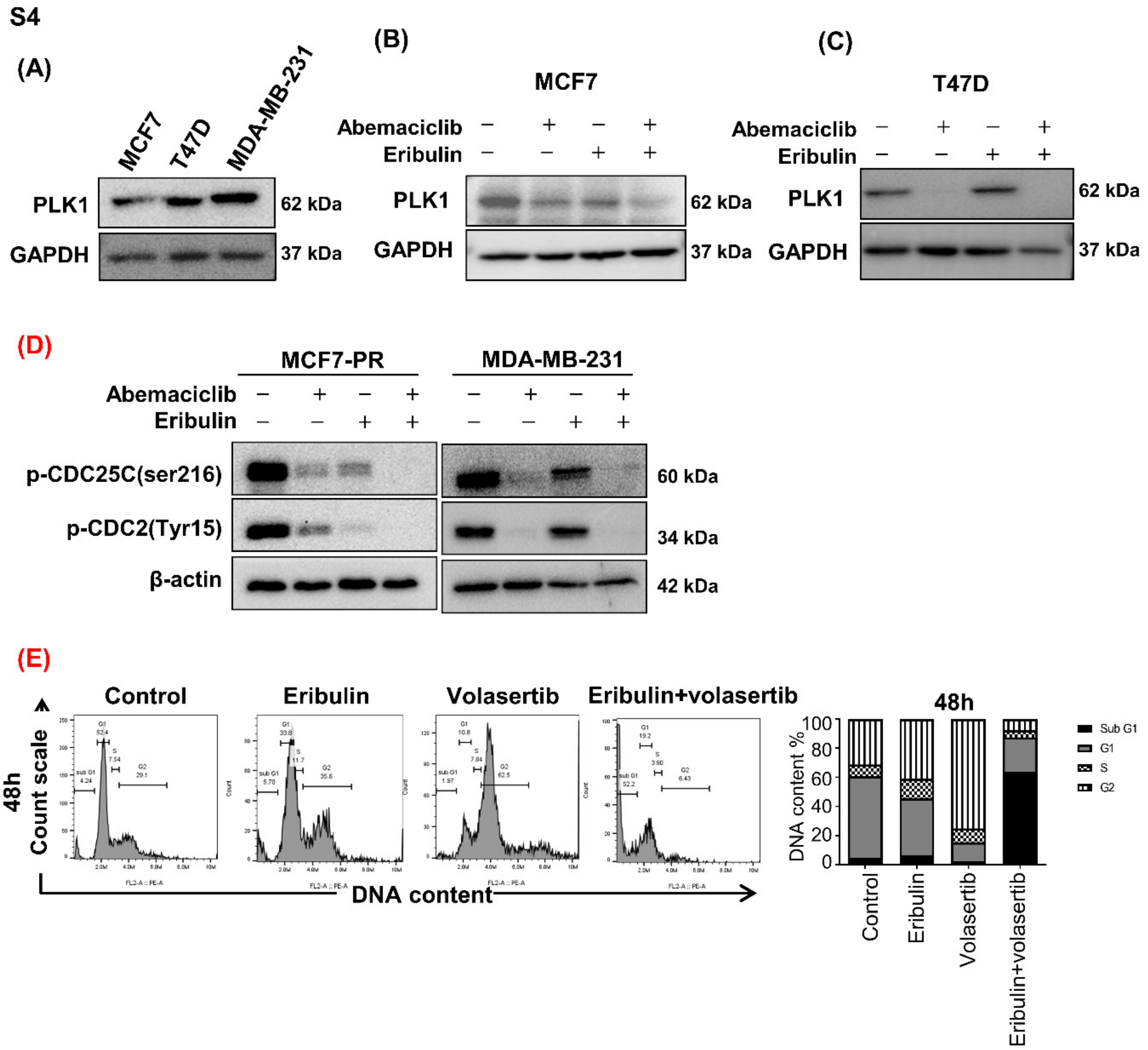
S4
(A)
MCF7
T47D
MDA-MB-231
PLK1
GAPDH
62 kDa
37 kDa
(B)
MCF7
Abemaciclib
Eribulin
PLK1
GAPDH
62 kDa
37 kDa
(C)
T47D
Abemaciclib
Eribulin
PLK1
GAPDH
62 kDa
37 kDa
(D)
MCF7-PR
MDA-MB-231
Abemaciclib
Eribulin
p-CDC25C(ser216)
p-CDC2(Tyr15)
β-actin
60 kDa
34 kDa
42 kDa
(E)
Control
Eribulin
Volasertib
Eribulin+volasertib
48h
Count scale
DNA content
DNA content %
Sub G1
G1
S
G2
Control
Eribulin
Volasertib
Eribulin+volasertib

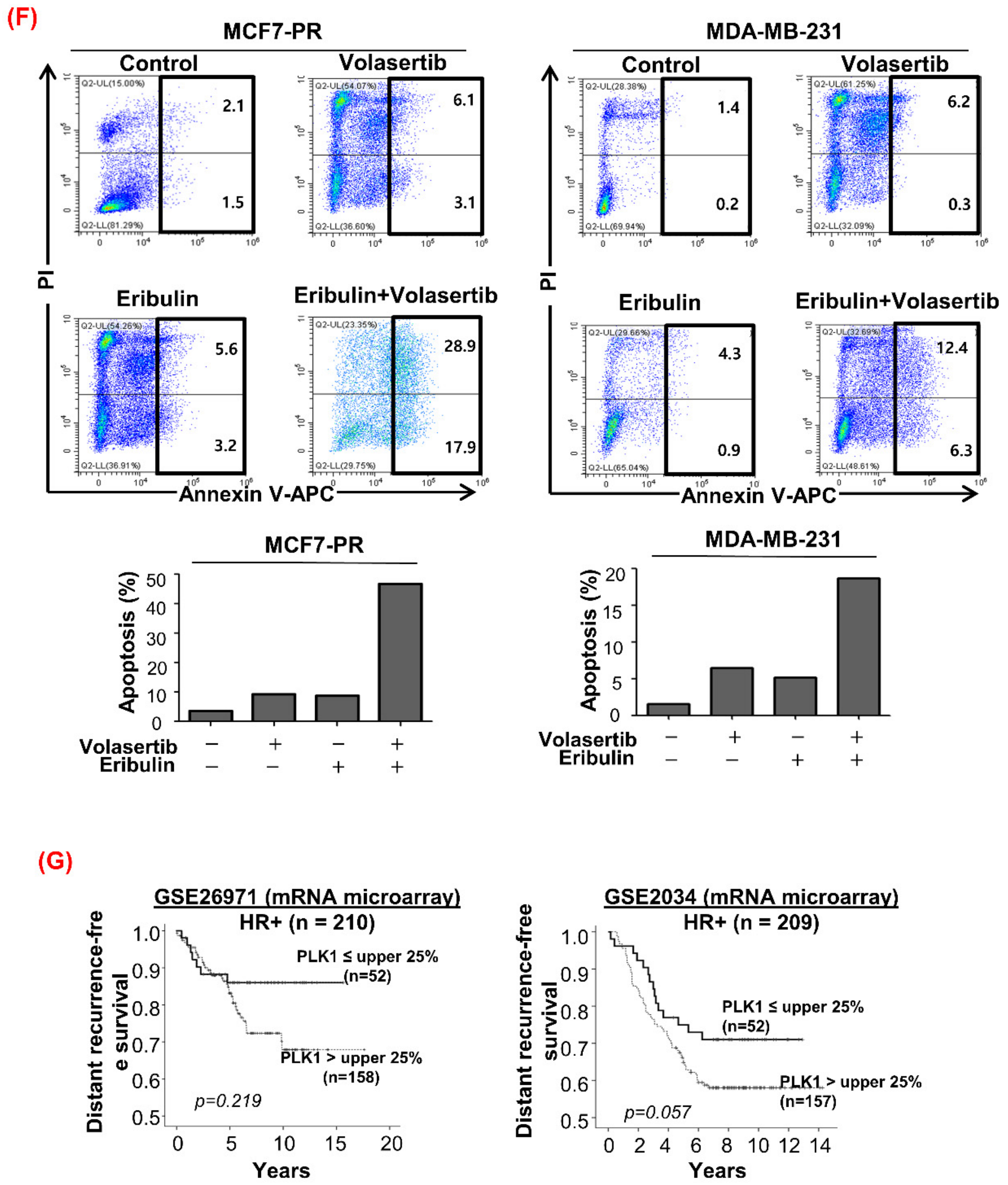


**Figure S4.** Abemaciclib enhances eribulin to inhibit PLK1 in the G2/M phase. (A) PLK1 expression in HR-positive cells (MCF7 and T47D) and TNBC (MDA-MB-231) cells by Western blot. (B–C) MCF7 and T47D cells were treated with IC50 concentration of eribulin or abemaciclib and sequential combination for 48 h. PLK1 expression and was analyzed by western blot. (D) MCF7-PR and MDA-MB-231 cells were treated with IC50 concentration of eribulin or abemaciclib and sequential combination for 48 h. pCDC2(Tyr15) and pCDC25C(ser216) expression was analyzed by western blot. (E) MCF7-PR cells were treated for 48h with IC25 concentration of eribulin or volasertib and their combination. Cell cycle distribution was analyzed by flow cytometry. (F) MCF7-PR and MDA-MB-231 cells were treated with IC25 concentration of eribulin or volasertib and their combination for 48 h. Cells were stained with annexin V-APC/PI, and the apoptosis was measured by flow cytometry. (G) Kaplan–Meier curves indicate distant recurrence-free survival according to relative PLK1 mRNA level from two independent public mRNA profiling data sets. High PLK1 expression predicts poor prognosis in HR-positive breast cancer patients.

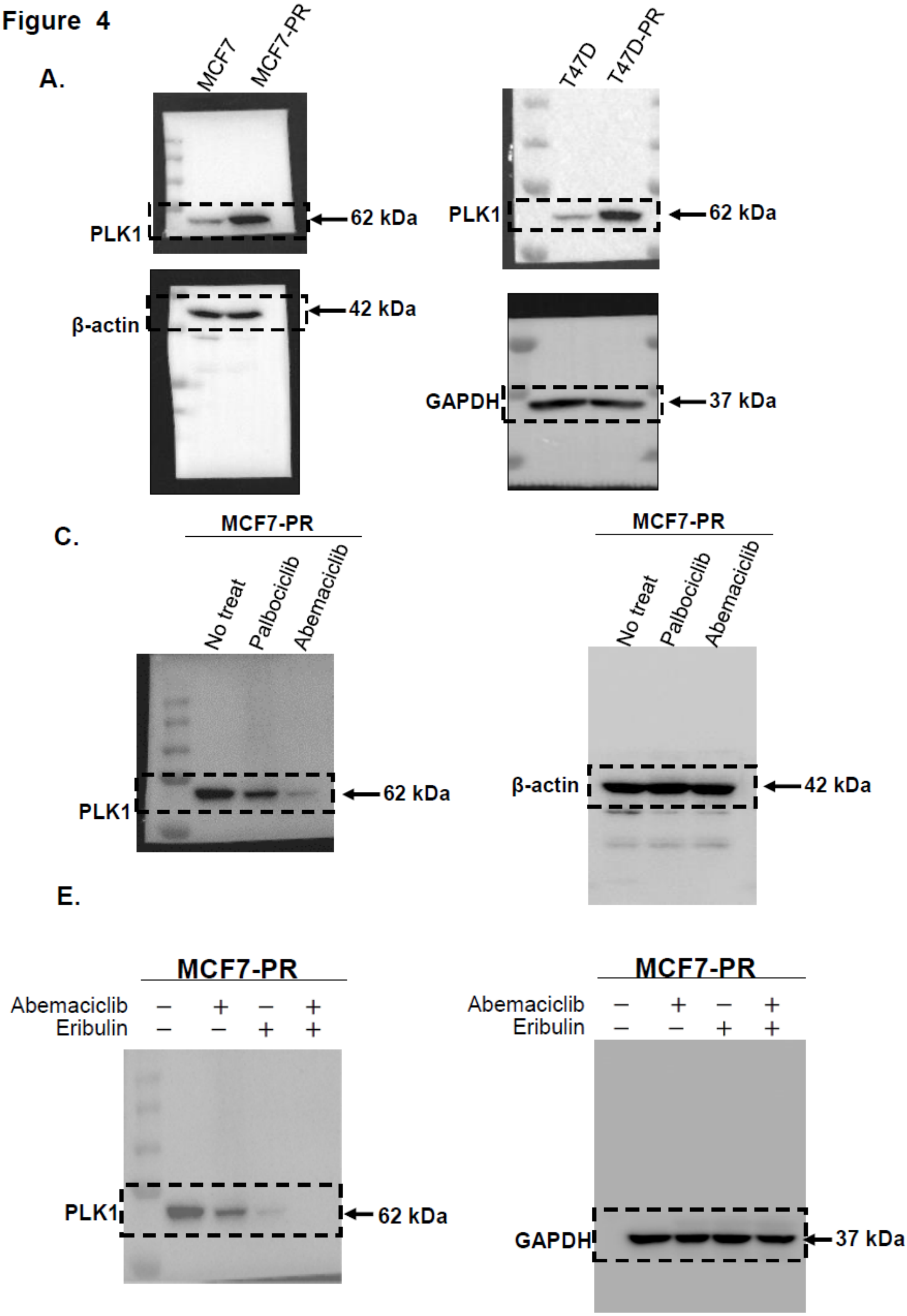
Figure 4
A.
MCF7
MCF7-PR
PLK1
62 kDa
β-actin
42 kDa
T47D
T47D-PR
PLK1
62 kDa
GAPDH
37 kDa
C.
MCF7-PR
No treat
Palbociclib
Abemaciclib
PLK1
62 kDa
MCF7-PR
No treat
Palbociclib
Abemaciclib
β-actin
42 kDa
E.
MCF7-PR
Abemaciclib − + − +
Eribulin − − + +
PLK1
62 kDa
MCF7-PR
Abemaciclib − + − +
Eribulin − − + +
GAPDH
37 kDa

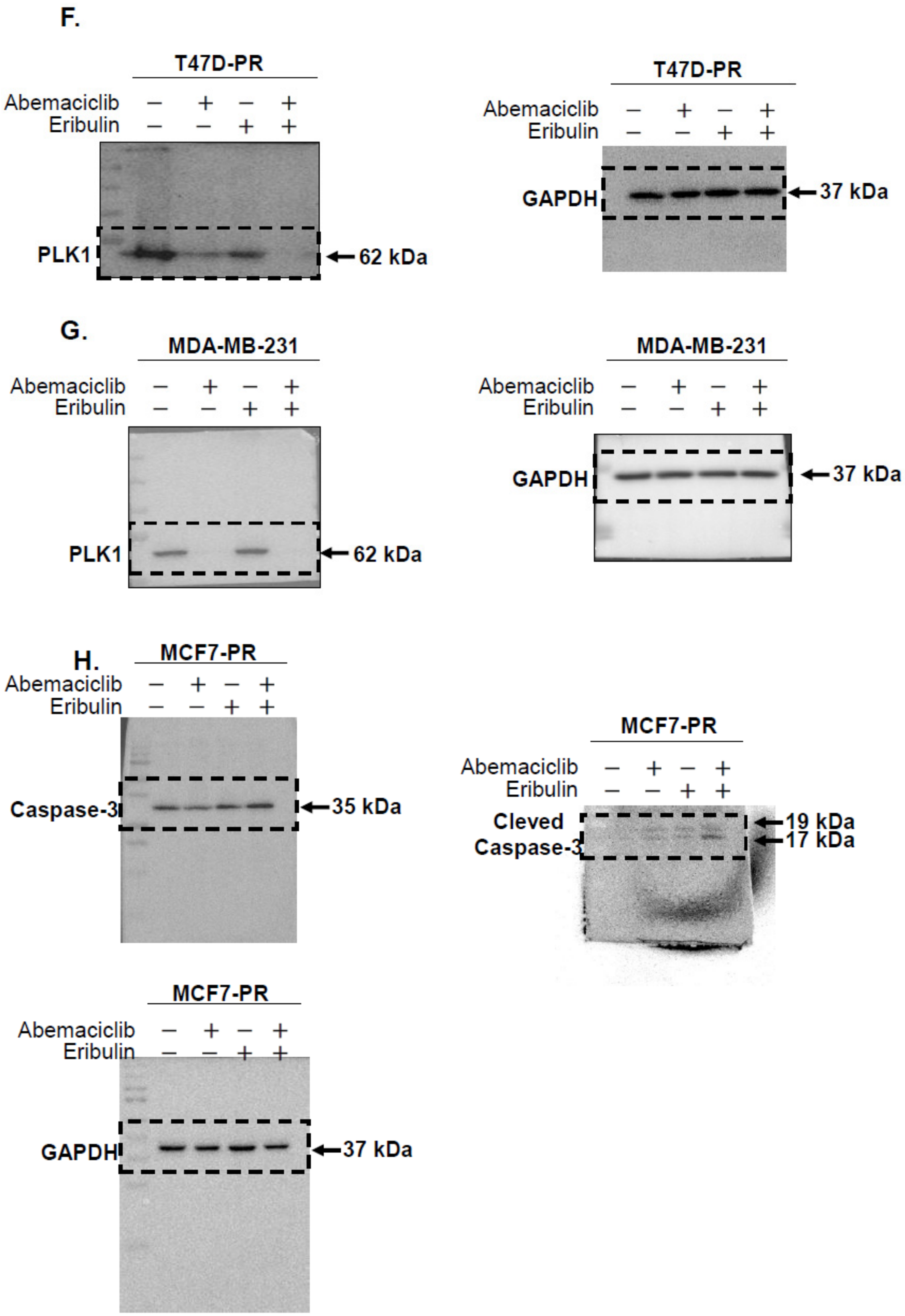
F.
T47D-PR
Abemaciclib – + – +
Eribulin – – + +
PLK1
62 kDa
T47D-PR
Abemaciclib – + – +
Eribulin – – + +
GAPDH
37 kDa
G.
MDA-MB-231
Abemaciclib – + – +
Eribulin – – + +
PLK1
62 kDa
MDA-MB-231
Abemaciclib – + – +
Eribulin – – + +
GAPDH
37 kDa
H.
MCF7-PR
Abemaciclib – + – +
Eribulin – – + +
Caspase-3
35 kDa
MCF7-PR
Abemaciclib – + – +
Eribulin – – + +
Cleved Caspase-3
19 kDa
17 kDa
MCF7-PR
Abemaciclib – + – +
Eribulin – – + +
GAPDH
37 kDa

# Figure 5

**F**

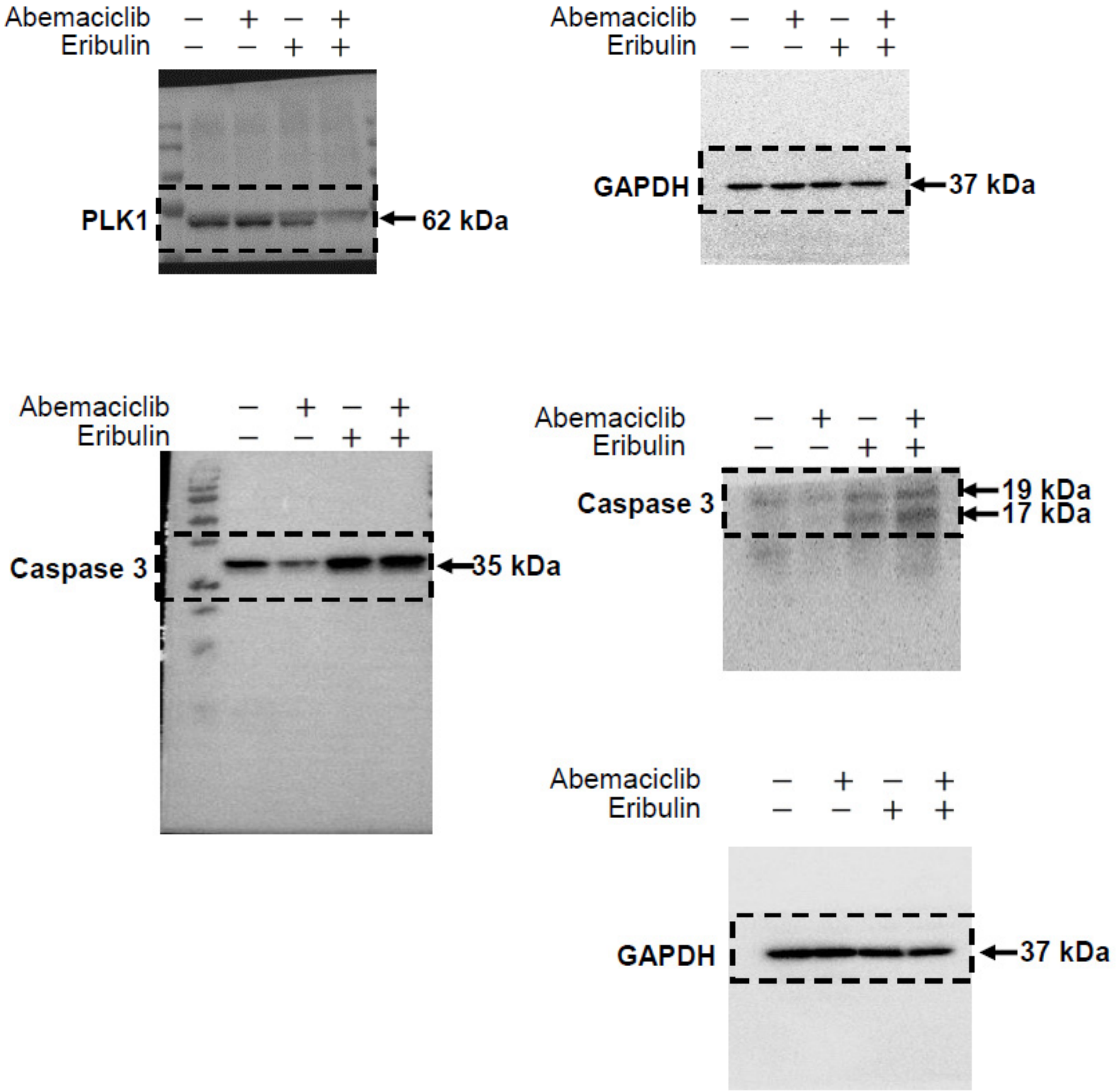
Abemaciclib – + – +
Eribulin – – + +
PLK1
62 kDa
Abemaciclib – + – +
Eribulin – – + +
GAPDH
37 kDa
Abemaciclib – + – +
Eribulin – – + +
Caspase 3
35 kDa
Abemaciclib – + – +
Eribulin – – + +
Caspase 3
19 kDa
17 kDa
Abemaciclib – + – +
Eribulin – – + +
GAPDH
37 kDa

# Figure S4

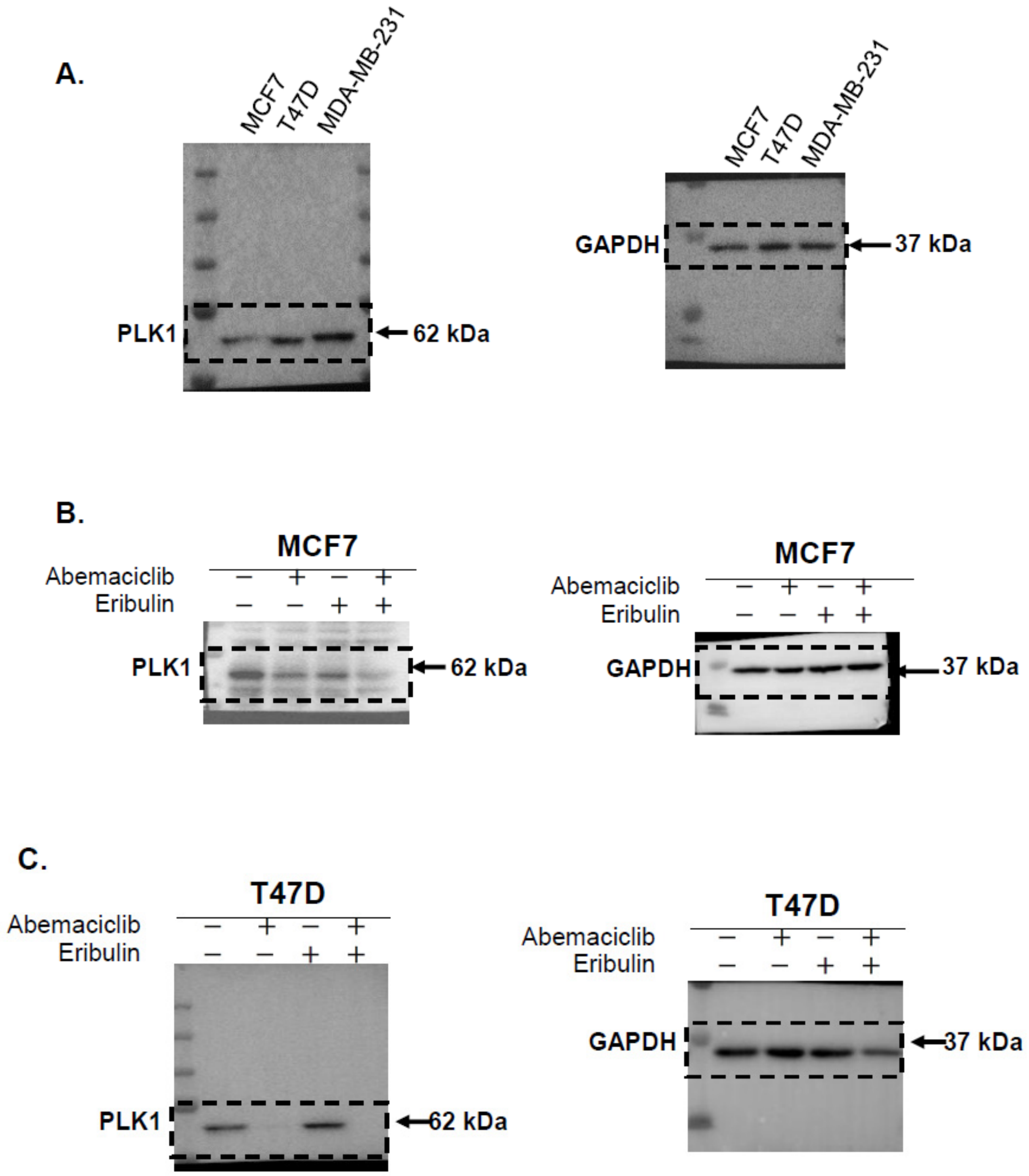
A.
MCF7
T47D
MDA-MB-231
PLK1
62 kDa
GAPDH
37 kDa
B.
MCF7
Abemaciclib
Eribulin
PLK1
62 kDa
MCF7
GAPDH
37 kDa
C.
T47D
Abemaciclib
Eribulin
PLK1
62 kDa
T47D
GAPDH
37 kDa

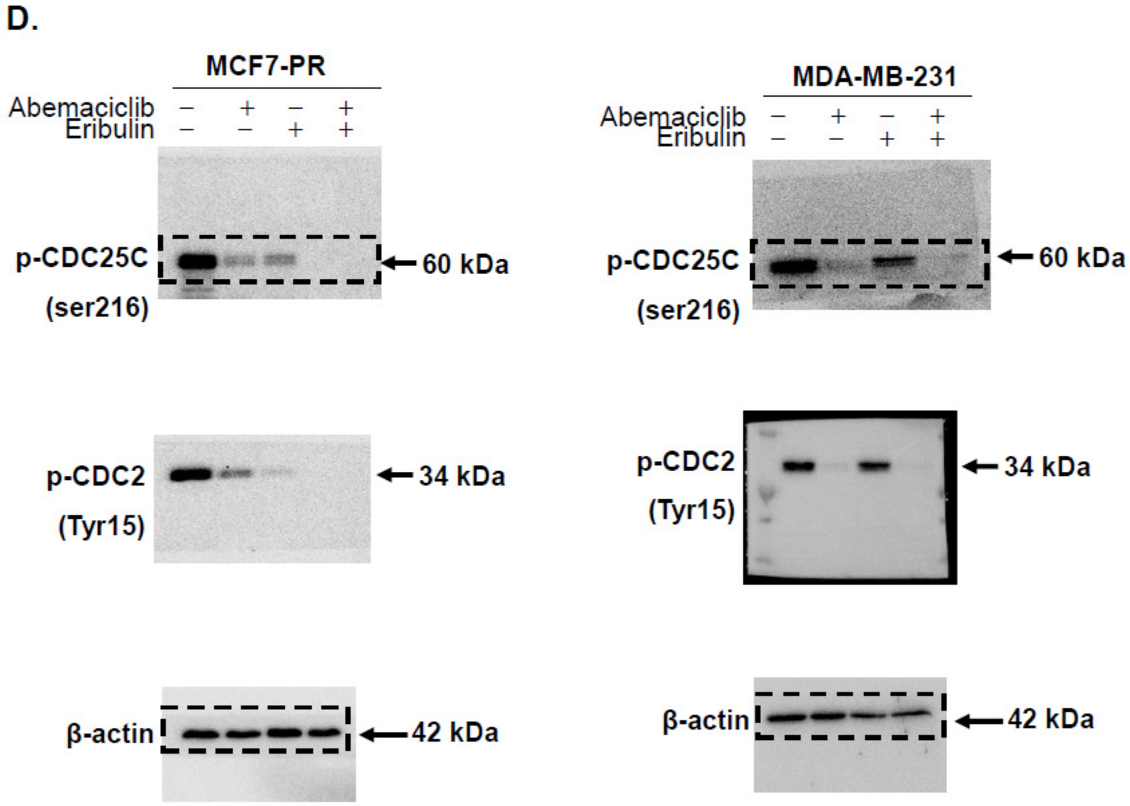


Figure S5: PDF file. Un-cropped immunoblot images.

**Supplementary Tables:**

**Supplementary Table S1**: Plasma concentration of the drugs applied to patients.

| Drugs | $C_{max}$ (ng/ml) | Mol.Wt (g/mol) | $C_{max}$ (μM) | References |
|---|---|---|---|---|
| Eribulin | 371 | 729 | 0.58 | [16] |
| Abemaciclib | 249 | 506.6 | 0.49 | [17] |
| Volasertib | 756 | 618.8 | 1.2 | [18] |

**Supplementary Table S2.** Primary and secondary antibodies used for western blot.

| Antibody | Host species | Dilution | Company |
|---|---|---|---|
| PLK1 | Rabbit | 1:1000 | Cell Signalling, Danvers, Massachusetts, USA |
| Caspase-3 | Rabbit | 1:1000 | Cell Signalling, Danvers, Massachusetts, USA |
| GAPDH | Rabbit | 1:1000 | LF-PA0212,AbFrontier, Seoul, Korea |
| Beta actin | Mouse | 1:5000 | Santa Cruz, Dallas, Texas, USA |
| Anti-Rabbit HRP | Goat | 1:5000 | Millipore, Burlington, Massachusetts, USA |
| Anti-Mouse HRP | Goat | 1:5000 | GeneTex, CA 92606, USA |

**Supplementary Table S3.** A list of 38 breast cancer cell lines in CCLE data base

| |
|---|
| BT-20 ,HCC70, BT-474, Hs 578T, BT-549, KPL-1, CAL-51,MCF7, CAMA-1, MDA-MB-134-VI, DU4475, MDA-MB-157, EFM-192A, MDA-MB-175-VII, EFM-19, MDA-MB-231, HCC1143, MDA-MB-361, HCC1187, MDA-MB-415, HCC1395, MDA-MB-436, HCC1419, |

| |
|---|
| MDA-MB-453, HCC1500, MDA-MB-468, HCC1569, SK-BR-3, HCC1806, T47D, HCC1937, UACC-812, HCC1954, UACC-893, HCC202, ZR-75-1, HCC2218, ZR-75-30 |

**Supplementary Table S4**. Patient characteristics in two public mRNA expression data sets

| Data set (patients no.) | GSE26971 (N=210) | GSE2034 (N=209) |
|---|---|---|
| HR | | |
| Positive | 210 | 209 |
| Negative | 0 | 0 |
| pTstage | | |
| T1 | 82 | NA |
| T2 | 122 | NA |
| T3 | 6 | |
| pNstage | | |
| N0 | 126 | NA |
| N1 | 46 | NA |
| N2 | 18 | NA |
| N3 | 20 | NA |
| LN metastasis | | |
| Yes | 126 | 209 |
| No | 84 | 0 |
| Distant recurrence | | |
| Yes | 44 | 80 |
| No | 166 | 129 |
| Median F/U (yr) | 6.21 | 7.17 |
| HR, Hormone Receptor; LN, Lymph Node; NA, Non-Available; F/U, Follow Up; yr, year; Staging is based on American Joint Committee on Cancer staging revised in 2002. | | |

**Supplementary Table S5**: Comparison of $IC_{50}$ concentration of palbociclib-resistant cells with their parental counterparts

| | MCF7 | MCF7-PR | *p*-Value | T47D | T47D-PR | *p*-Value |
|---|---|---|---|---|---|---|
| Palbociclib $IC_{50}$ (μM) | 0.75 | 7.15 | <0.001 | 0.26 | 3.37 | 0.001 |
| Ribociclib $IC_{50}$ (μM) | 0.59 | 2.04 | 0.044 | 0.46 | 4.73 | <0.001 |
| Abemaciclib $IC_{50}$ (μM) | 0.73 | 3.4 | 0.038 | 0.77 | 4.8 | 0.003 |

**Supplementary Table S6**: Toxicity and half-life profile of CDK4/6 inhibitors

| Drug | Toxicity of G3-4 | Elimination half-life [24] | Reference |
|---|---|---|---|
| Palbociclib | Neutropenia 65% | 29 hours | [25] |
| Ribociclib | Neutropenia 46 % | 32 hours | [26] |
| Abemaciclib | Neutropenia 21 % | 18.3 hours | [23] |

**Supplementary Table S7.** Completed or ongoing clinical trials of combined taxanes and CDK4/6 inhibitor.

| **Completed or ongoing clinical trial of combined taxanes and CDK4/6 inhibitor**. | | | | | |
|---|---|---|---|---|---|
| **Author (NCT number)** | **Study status** | **Drug** | | **Phase** | **Disease** |
| | | **Taxane** | **CDK4/6 inhibitor** | | |
| NCT02501902 | Completed (2018) | nab-paclitaxel | Palbociclib | 1 | Metastatic pancreatic ductal adenocarcinoma |
| NCT01320592 | Completed (2019) | Paclitaxel | Palbociclib | 1 | Metastatic breast cancer |
| NCT04594005 | Not yet recruiting | Paclitaxel | Abemaciclib | 1/2 | CDK4/6 pathway activated tumors |
| NCT02608216 | Recruiting | Paclitaxel | Ribociclib | 1 | Metastatic breast cancer |

| | | | | | |
|---|---|---|---|---|---|
| NCT02599363 | Active, not recruiting | Paclitaxel | Ribociclib | 1 | Metastatic breast cancer |
| NCT03056833 | Active, not recruiting | Carboplation+Paclitaxel | Ribociclib | 1 | Platinum-sensitive ovarian cancer |
| NCT02494921 | Active, not recruiting | Docetaxel | Ribociclib | 1/2 | Castration-resistant prostate cancer |
